# Editorial: Bilingual Language Development: The Role of Dominance

**DOI:** 10.3389/fpsyg.2019.01064

**Published:** 2019-05-21

**Authors:** Cornelia Hamann, Esther Rinke, Dobrinka Genevska-Hanke

**Affiliations:** ^1^Institute for English and American Studies, University of Oldenburg, Oldenburg, Germany; ^2^Institute of Romance Languages and Literatures, Goethe-Universität Frankfurt am Main, Frankfurt, Germany

**Keywords:** bilingualism, language acquisition, dominance, L1 attrition, dominance operationalization

It has long been established that bilingual speakers are rarely balanced in their languages so that one language is dominant. The contributions to Bilingual Language Development: The Role of Dominance focus on the potential effects of language dominance on the competence and processing of bilinguals, covering a large variety of language combinations and domains. Important aspects of such work are the interplay of L1-maintenance/attrition and possible L2-dominance, the direction of cross-linguistic influence (CLI) or code-mixing, as well as the effects of bilingualism on cognitive development, each addressed in several contributions. However, such research presupposes a definition of dominance, which is far from being settled. This gives rise to considerable differences in the operationalization of the concept across studies. Among other factors, many researchers use proficiency to determine dominance (Genesee et al., 1995), others exposure and use (Argyri and Sorace, 2007), or environmental language (Polinsky, 2008, but see Schmeißer et al., 2015). Recently, a trend developed toward an integrated perspective (Birdsong, 2018). In their overview, Cantone et al. (2008) argue for a definition combining experiential with performance factors (see also Montrul, 2015 and Silva-Corvalán and Treffers-Daller, 2016) while Bedore et al. (2012) demonstrate that experiential measures and relative proficiency are related. More specifically, Unsworth (2016) argues that experiential variables predict performance/proficiency, so that relative amount of exposure and use can serve as a proxy for language dominance. Complementing this work, Unsworth et al. estimate relative exposure and use with a parental questionnaire and obtain performance measures through spontaneous speech production as well as standardized vocabulary measures. Their results indicate that language use is a stronger predictor of proficiency than exposure, pointing to its importance for dominance.

These findings are crucial for research on language impairments. For developmental language disorder (DLD) it is recommended to test children in both languages or at least in their dominant language and adjust norms according to dominance (Thordardottir, 2015) while therapy for aphasia should be conducted in the dominant language. Three studies in this collection address language impairments. Abed Ibrahim and Fekete investigate bilingual children with German as early-L2. They operationalize dominance through experiential factors and investigate performance in two repetition tasks. Using partitioning around medoids, which does not rely on a priori group assignment, they show that dominance influences performance in typical bilinguals without negatively affecting diagnostic accuracy. Their findings suggest that testing in the majority language does not necessarily disadvantage bilingual children. Meir compares the morpho-syntactic abilities of four groups of Russian-German bilingual children: children with a weaker language, balanced bilinguals and children dominant in that language, as well as bilingual children with DLD. While error patterns are the same across typical bilingual groups, unbalanced bilinguals used complex syntax, relying on resources from the dominant language. However, bilinguals with DLD simplified structures. This suggests that the non-dominant language of unbalanced typical bilinguals may be delayed, not deviant, whereas acquisition patterns of bilinguals with DLD are distinct, resembling those of monolingual children with DLD. While there is an extensive body of research on bilingual children with DLD, studies on bilingual patients with neurodegenerative diseases are much rarer. Karpathiou et al. thus provide a valuable study of the factors determining language preservation. They show that lexical and grammatical abilities of a bilingual with L1-Greek and late L2-French are impaired in both languages, with better preservation of the dominant language.

CLI and language mixing provide more evidence for the importance of a dominance definition. Investigating the acquisition of residual V2-structures in English by three Norwegian-English simultaneous bilingual children, Andersson and Bentzen find different patterns of CLI, but argue that these differences cannot be attributed to dominance. They also discuss whether dominance determines the direction of mixing (Genesee et al., 1995) arguing that dominance, when determined by proficiency and experiential measures, does not affect mixing. Also focussing on CLI, Puig-Mayenco et al. examine the role of dominance for the competence of children with L1-Spanish/early-L2 Catalan, and children with L1-Catalan/early-L2 Spanish. Focusing on one property of Catalan, the occurrence of pre-verbal Negative Concord Items (NCIs) not licit in Spanish, and one of Spanish more restricted in Catalan, Differential Object Marking (DOM), the authors observe CLI for DOM but not for NCIs. L1-Spanish/L2-Catalan speakers over-accept DOM in Catalan, suggesting that their dominance in Spanish influences the directionality of CLI, which the performance of the L1-Catalan/L2-Spanish group confirms.

It has been observed that language dominance may shift during development, and Gagarina and Klassert investigate the L1-competence of bilingual children in such a dominance-shift context: the systematic exposure to L2 in kindergarten/preschool. They examine experiential factors, such as input provided by the nuclear family, as predictors for lexical and (verbal/nominal) morphological development in L1 after change of input dominance. Interestingly, age, gender, and L2-AoO all differently impact these domains. Importantly, verbal inflection proves to be more robust than case inflection. The latter finding ties in with the longitudinal study of Schulz and Grimm investigating experiential measures and, crucially, timing in acquisition as factors influencing development of the majority language. The study compares simultaneous to early-L2 bilingual children and shows that while age of onset affects early-acquired phenomena such as subject-verb-agreement, this is not the case for late-acquired phenomena such as case marking. Clearly, timing modulates age effects. Dominance, determined by experiential measures, does not play a role for early or late acquired phenomena in simultaneous bilinguals.

Language dominance and attrition are interrelated, established with similar measures and influenced by the same factors so that they might represent two stages of the same phenomenon as suggested by Köpke and Genevska-Hanke. Among several studies on L1-attrition, Schmid and Yilmaz offer an integrated perspective investigating the role of various experiential and proficiency predictors of language dominance in four migrant populations. Focusing on dominance shifts and factors facilitating L1-attrition/maintenance, they suggest that different aspects of bilingualism affect language development in different ways. For L1, measures of informal language use play a role in determining whether a bilingual is a good maintainer, while success in L2-acquisition depends heavily on personal factors such as the educational level. Similarly, Montrul et al. find effects of exposure and use on the knowledge of Hindi-case marking in Hindi-English bilinguals with different dominance patterns: balanced bilinguals in India outperform unbalanced L2 and heritage speakers in the US, who show instability in their production/knowledge of the Hindi case system. In contrast, the study of Mitrofanova et al. finds that a combination of experiential and proficiency measures is the best predictor for different attrition/maintenance patterns. Studying the acquisition of gender in Russian by Russian-Norwegian bilingual children in Norway they show that bilingual and monolingual children were sensitive to phonological gender cues, albeit to different degrees.

Whereas the previous studies reveal specific domains as problematic for L2-acquisition or L1-maintenance in different experiential situations, Caloi et al. provide a broader view. They compare adult heritage speakers of Italian, late L2-learners with L1-Italian, and Italian monolinguals as to the strategy employed when answering new information questions. Monolinguals prefer a Verb-Subject-structure, whereas heritage and L2-speakers behave alike, but different from monolinguals, in opting for Subject-Verb-answers. These group comparisons lead the authors to remind the reader that a “bilingual speaker is not two monolinguals in one” (Grosjean, 1989)—the grammatical features of L1 are well-mastered and it is the richer experience which leads to a different, a wider, not an attrited or incomplete system.

Apart from the language domains studied so far, the use of null- and overt subjects has long been in the focus of research on the effects of language dominance on L2-development and L1-maintenance, and two of the contributions address this domain. Di Domenico and Baroncini look at the role of dominance and age of onset on the choice of overt and null pronominal subjects in native and near-native speakers of Greek and Italian. The results reveal age of onset as a factor: near-natives overuse overt pronouns, simultaneous bilinguals do not. Interestingly, effects of dominance are found for null pronouns and lexical DPs but not for overt pronouns.

Turning to psycholinguistic aspects of bilingualism and language dominance, Köpke and Genevska-Hanke define dominance as the relative accessibility of each of the languages of a bilingual for language processing. Taking late L1-attrition and dominance to represent different stages of a continuum, they investigate knowledge of pronominal subjects (see also Di Domenico and Baroncini) in a speaker of (pro-drop) L1-Bulgarian and (non-pro-drop) L2-German, who had late L2-onset and fairly long residency in Germany. The authors argue that attrition of a highly-entrenched L1 affects language processing not underlying representations, and that it does so temporarily only, disappearing fast after a limited re-exposure to L1-input. This opens the question if re-exposure to Italian would have changed the performance of the bilinguals in the study by Caloi et al..

Regarding notions of language accessibility, an advantage in cognitive flexibility has been attributed to bilinguals (Bialystok, 2005), but studies have not always reached the same conclusions. Nicoladis et al. contribute to the discussion whether the need to access each language depending on context accounts for flexibility by investigating whether balanced bilinguals have an advantage over other bilinguals. Study of French-English bilingual children shows that none of the experiential or proficiency measures predicts cognitive flexibility. Also focusing on cognitive aspects, Altman et al. investigate the influence of dominance on metalinguistic awareness, MA, and whether MA mediated by dominance influences vocabulary size. Crucially, only the Hebrew vocabulary size of the Russian-dominant children is affected by MA. They rely on their fast mapping abilities to expand vocabulary, which, the authors argue, reflects their level of vocabulary development in the societal language.

The studies in this volume present a multifaceted picture of the role of language dominance for L1-maintenance/attrition, L2-development and CLI. Though a unified story cannot emerge for such a complex subject, interesting new venues are explored including the impact of dominance shift during L1-re-exposure, comparisons of different types of bilingual groups, or operationalization of dominance through experiential measures. The variety of approaches and results is in part owed to the many language combinations studied and the fact that bilingual children, adults and atypical speakers are investigated. This diversity constitutes the interest of this volume.

## Author Contributions

All authors listed have made a substantial, direct and intellectual contribution to the work, and approved it for publication.

### Conflict of Interest Statement

The authors declare that the research was conducted in the absence of any commercial or financial relationships that could be construed as a potential conflict of interest.
